# PSFD-Musa: A dataset of banana plant, stem, fruit, leaf, and disease

**DOI:** 10.1016/j.dib.2022.108427

**Published:** 2022-06-28

**Authors:** Epsita Medhi, Nabamita Deb

**Affiliations:** Department of Information Technology, Gauhati University, Guwahati, Assam 781014, India

**Keywords:** Plant classification, Disease classification, Image processing, Sigatoka disease, Banana aphid

## Abstract

In recent times, the classification and identification of different fruits and food crops have become a necessity in the field of agricultural science; for sustainable growth. Probable processes have been developed worldwide to improve the production of food crops. Problem-specific, clean and crisp datasets are also lagging in the sector. This article introduces an image dataset of varieties of banana plants and the diseases related to them. The varieties of Banana plants that we have considered in the dataset are the Malbhog (**Musa assamica**), Jahaji (**Musa chinensis**), Kachkol (**Musa paradisiaca L.**), Bhimkol (**M. Balbisiana Colla**). And the diseases and pathogens that we have considered here are the Bacterial Soft Rot, Banana Fruit Scarring Beetle, Black Sigatoka, Yellow Sigatoka, Panama disease, Banana Aphids, and Pseudo-Stem Weevil. A dataset of Potassium deficiency has been also considered in this article. A total of 8000+ processed images are present in the dataset. The purpose of this article is to provide the Researchers and Students in getting access to our dataset that would help them in their research and in developing some machine learning models.

## Specifications Table

**Table:** 

Subject	Agronomy, Horticulture
Specific subject area	Image Processing, Machine Learning
Type of data	Images of different varieties of banana plants that include the stem, leaf, and fruit images. Images of different diseases that affect the banana plants and also the deficiency of the plant.
How the data were acquired	Raw RGB images of the leaves, stems, and fruits of banana plants were captured under natural light with a mobile phone camera Samsung SM-G610F having 9.6 megapixels and with Nikon SX 70 having 18.3 megapixels. While the images were captured it was taken into consideration that an average light falls on the images.
Data format	Raw images having the format of .jpg.
Description of data collection	The images present in the dataset are collected manually using a good quality mobile phone and a DSLR camera, under bright sunlight, but some of them even fall under the shaded parts of the plant. The images were collected from different Banana plantation fields containing the images of stems, leaves, fruits, and flowers of the plant and some common diseases that affect the plant. The images were captured randomly and were sorted with the help of an expert. The images had the original dimension to be 3096 × 4128 and it was resized again to the dimension of 256 × 256. Our proposed dataset can be used by Researchers and Students of different backgrounds to train, test, and validate classification models.
Data source location	• BORTARI VILLAGE, Chaygaon, Kukurmara, District – Kamrup (Rural), Assam, India. • HAJO VILLAGE, District – Kamrup (Rural), Assam, India.
Data accessibility	Data is available at Mendeley Data, under the DOI: 10.17632/4wyymrcpyz.1https://data.mendeley.com/datasets/4wyymrcpyz/1

## Value of the Data

• The dataset provided here is the collection of different varieties of banana plants, some common diseases that affect them, and their deficiency. These varieties of banana plants are indigenously found in Assam. The data can be useful in the way to classifying the different diseases and pathogens which are harmful to the banana plantations. It is also found to be useful to study the status of healthy plants.
• As the PSFD-Musa dataset consists of 8000+ processed images, therefore it would be beneficial for the researchers, students, and any other knowledge learning inquiries just in the case of machine learning, image processing, computer vision, deep learning, etc.
• The people who regularly monitor the phytopathology of plants and apply their work for research-related demonstration and application can systematically extract and use the data provided here.
• With the help of this dataset, one can do the process of Calibration of the data as required and also can validate it. Accuracy comparison between models can also be done with these datasets.
• The classification of the dataset can be very useful for people who are related to the Agri Industry and also for the customers, fruit vendors, companies related to the export of fruits, and many more.
• Development of a new system can also be possible with these data.

## 1 Data Description

Banana plants (Musa spp.) [6] is a globally distributed fruit crop and is considered to be the largest herb [1]. It develops from the rhizome to form the stem, and the leaves grow larger. It does not form any branches like other trees, whereas the leaves form the branches at the shoot apex. Flowers develop at the apical meristem itself and from that, a bunch of the fruits develop. Varieties of banana plants can be found worldwide. It grows in Tropical regions and requires a hot and humid climate to develop itself [2].

**Fig. 1:**
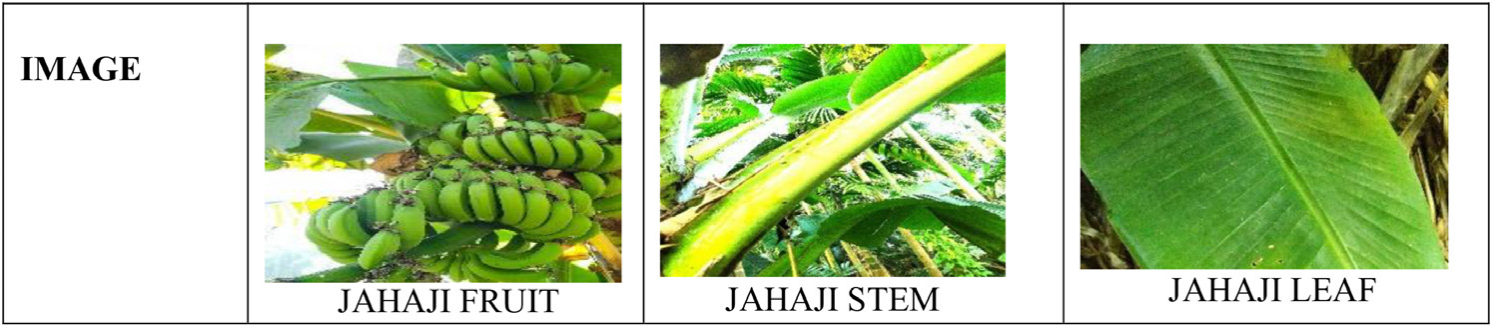
Sample of images from the Jahaji banana (Musa chinensis) dataset.

**Fig. 2:**
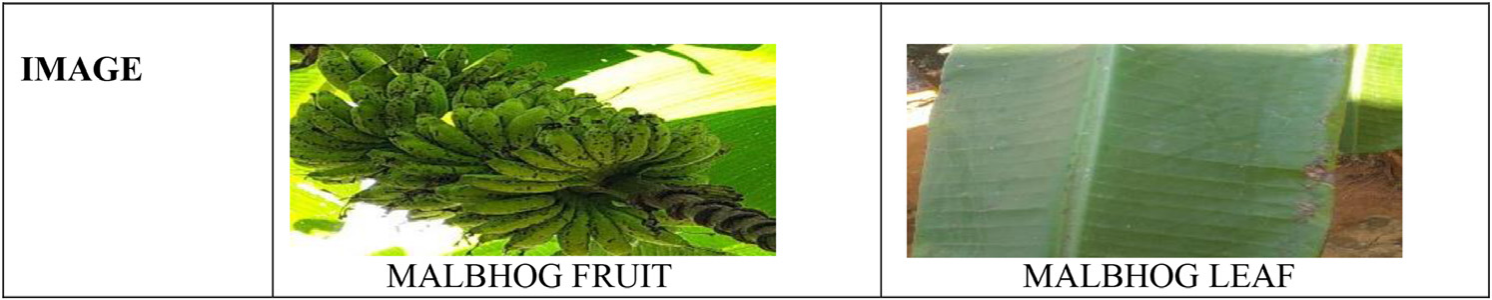
Sample of images from the Malbhog banana (Musa assamica) dataset.

**Fig. 3:**
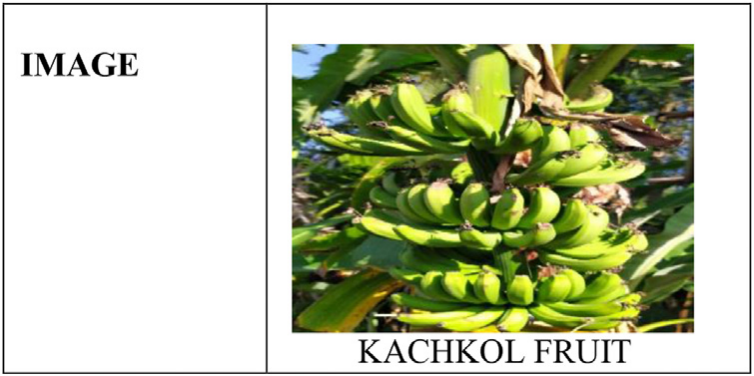
Sample of an image from the Kachkol Banana (Musa paradisiaca L.) dataset.

**Fig. 4:**
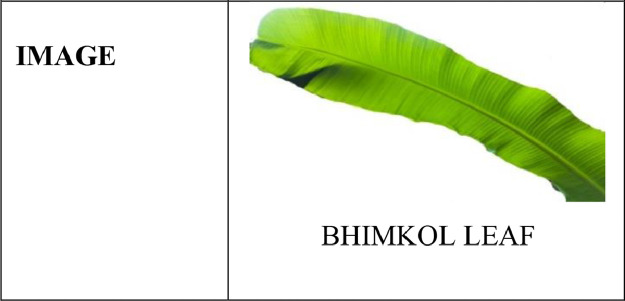
Sample of an image from the Bhimkol Banana (M. Balbisiana Colla) dataset.

**Fig. 5:**
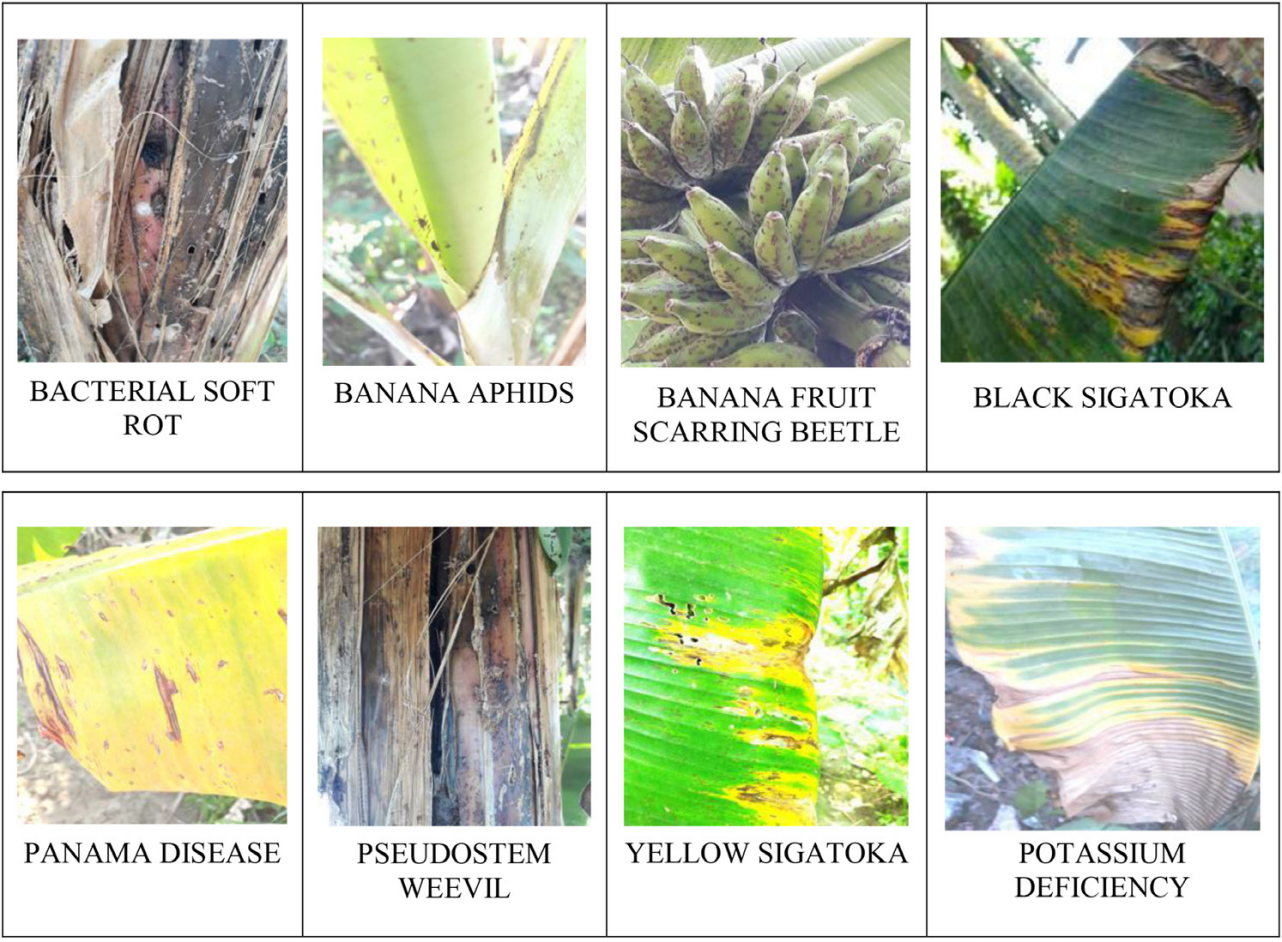
Sample of diseases that affects the banana plants and also the deficiency that incurs production loss in the banana plants.

It is seen that each part of a banana plant can get infected with different types of bacterial, fungal, and viral diseases [3,5]. Out of which many of them are dangerous diseases that affect it and its production. Deficiency diseases too can incur a heavy loss over the banana plantations. To get familiarized with different varieties of banana plants and to know some of the common diseases that affect the plants, we have created a PSFD-Musa DATASET, for the banana plants that are indigenously found in different parts of Assam. The dataset is divided into 3 subfolders. The first folder comprises the images of different varieties of banana plants which further consists of 7 classes namely Malbhog fruit (Musa assamica), Malbhog leaf (Musa assamica), Jahaji fruit (Musa chinensis), Jahaji stem (Musa chinensis), Jahaji leaf (Musa chinensis), Kachkol fruit (Musa paradisiaca L.), Bhimkol leaf (M. Balbisiana Colla). Samples of each class have been shown in Fig. 1, Fig. 2, Fig. 3, Fig. 4. The second folder comprises different diseases that affect the banana plants which again comprises 7 classes namely: Bacterial Soft Rot, Banana Fruit Scarring Beetle, Black Sigatoka, Yellow Sigatoka, Panama disease, Banana Aphids, and PseudoStem Weevil. And the last folder is of the deficiencies [4] that hamper the plants which are of 1 class namely: Potassium deficiency. Samples of banana diseases and deficiency have been shown in Fig. 5. The images provided here are raw as well as processed data and are in the format of .jpg. The dataset has every possibility to be used in the classification process and also can be used as a machine learning model.

## 2 Experimental Design, Materials and Methods

In this section of experimental design, materials, and methods, all the pre-processing steps applied to the data are mentioned to get the resulting dataset.

### 2.1 Experimental Design and Materials

The images of the dataset have been acquired from villages in and around Guwahati, Assam, India. The Cameras that were used to acquire the images were the Samsung J7 SM-G610F mobile phone camera of 9.6 megapixels and the Nikon SX 70 of 18.3 megapixels. The images have been captured manually under bright sunlight whereas some of the images have fallen under the shaded portion away from the sunlight.

### 2.2 Methods

For our PSFD-Musa DATASET, raw images were collected from the different banana plantations of Assam. The images were captured using a mobile phone camera and a digital camera under different lighting conditions. The image data are in the RGB and the .jpg format. The images are separated into their different varieties and kept separated concerning their Stem, Leaf, and Fruit. Banana plant images are then subdivided into different folders depending upon the diseases that affect them. Originally the images were of 3096 × 4128 dimensions. It was resized into 256 × 256 dimensions using Python programming so that the processing becomes easier. Because the raw images were very less in number therefore we had to augment the images and the dataset now consists of more than 8000 image data. Augmentations had been performed using Python programming language. Because of the lack of data, we got only 1 class of deficiency in the Banana pants. The images have been verified by different agricultural experts, horticulturists, and banana plantation farmers. The following tables, that is Table 2 gives a detailed description of the Image specification concerning varieties of fruit. Table 3 describes the image specification of the diseases and Table 4 describes the image specification of the deficiency in banana plants.

**Table 1:** Steps of data acquisition have been described in the tabular form.

Sl. No.	Process	Time	Work
1.	Image capture	April to June **(2021)**	The images were acquired under bright sunlight and some are acquired under
			The shaded part of the plant.
2.	Preparation of the Dataset	After July	Original dimension of the images i.e. 3096 × 4128 was resized into the dimension 256 × 256 and the images were classified into different folders.

**Table 2:** Image specification concerning varieties of fruit.

		Varieties of Banana Plant (Image Data)				
Sl. No.	Properties	Malbhog	Jahaji	Kachkol	Bhimkol	Total
1.	LEAF IMAGES	1605	336	-	402	2343
2.	STEM IMAGES	-	102	-	-	102
3.	FRUIT IMAGES	144	42	30	-	216
4.	DIMENSION	(256 × 256)	(256 × 256)	(256 × 256)	(256 × 256)	
5.	HORIZONTAL RESOLUTION	96 dpi	96 dpi	96 dpi	96 dpi	
7.	VERTICAL RESOLUTION	96 dpi	96 dpi	96 dpi	96 dpi	
8.	BIT DEPTH	24	24	24	24	
					TOTAL	2,661

**Table 3:** Image specification concerning some common diseases.

		Varieties of Diseases (Image Data)		
Sl. No.	Diseases	Images	Dimension	Resolution
1.	Bacterial Soft Rot	1078	(256 × 256)	96 dpi
2.	Banana Aphids	366	(256 × 256)	96 dpi
3.	Banana Fruit Scarring Beetle	150	(256 × 256)	96 dpi
4.	Black Sigatoka	474	(256 × 256)	96 dpi
5.	Panama Disease	102	(256 × 256)	96 dpi
6.	Pseudo stem Weevil	2736	(256 × 256)	96 dpi
7.	Yellow Sigatoka	264	(256 × 256)	96 dpi
	TOTAL	5,170

**Table 4:** Image specification concerning deficiency.

		Nutrition Deficiency (Image Data)		
Sl. No.	Deficiency	Images	Dimension	Resolution
1.	Potassium	1530	(256 × 256)	96 dpi
	TOTAL	1,530		

## Ethics Statements

The work presented here neither involves any human subjects nor any animal experiments. It consists of all the self-acquired images and does not contain any images collected from social media platforms. We did not receive any funds for carrying out this work.

## CRediT authorship contribution statement

**Epsita Medhi:** Conceptualization, Methodology, Data curation, Investigation, Writing – original draft, Validation. **Nabamita Deb:** Supervision, Writing – review & editing.

## Declaration of Competing Interest

The authors declare that they have no known competing financial interests or personal relationships that could have appeared to influence the work reported in this paper.

## Data Availability

PSFD-Musa DATASET (Original data) (Mendeley Data).
